# Rerefinement of poly[di­aqua­bis­(μ_3_-2-methyl­pro­pano­ato-κ^4^ *O*:*O*,*O*′:*O*′)bis­(μ_3_-2-methyl­propano­ato-κ^3^ *O*:*O*:*O*)(μ_2_-2-methyl­propano­ato-κ^3^ *O*:*O*,*O*′)(2-methyl­propano­ato-κ^2^ *O*,*O*′)trilead(II)]

**DOI:** 10.1107/S2414314620013115

**Published:** 2020-10-06

**Authors:** Erika Samolová, Jan Fábry

**Affiliations:** a Inst. of Physics of the Czech Academy of Sciences, Na Slovance 2, 182 21 Praha 8, Czech Republic; Vienna University of Technology, Austria

**Keywords:** crystal structure, hydrogen bonding, carboxyl­ates, the Cambridge Structural Database

## Abstract

The crystal structure of the title complex, [Pb_3_(C_4_H_7_O_2_)_6_(H_2_O)_2_]_*n*_, was redetermined on basis of modern CCD-based single-crystal X-ray data at 120 K. The current study basically confirms the previous report [Fallon *et al.* (1997 ▸). *Polyhedron*, **16**, 19–23] at 190 K, but with higher accuracy and precision. In particular, positional disorder of one of the 2-methyl­propano­ate anions over two sets of sites was resolved, showing a refined ratio of the disorder components of 0.535 (9):0.465 (9). The three independent cations in the structure have coordination numbers of [7 + 1], [6 + 1], and [5 + 3], with O atoms belonging either to carboxyl­ate groups or water mol­ecules. This arrangement leads to the formation of sheets parallel to (  01), whereby the hydro­phobic 2-methyl­propanyl groups of the anions are oriented above and below the hydro­philic sheets to form a layered structure. Within a sheet, hydrogen bonds of the type O_water_—H⋯O are formed, whereas the hydro­phobic groups between adjacent layers inter­act through van der Waals forces.

## Abstract 2

The crystal structure of [Pb_3_(C_4_H_7_O_2_)_6_(H_2_O)_2_]_*n*_ was redetermined, revealing details of the disorder of one of the hydro­carbon chains in one of the six independent 2-methyl­propano­ate anions.

## Graphical abstract

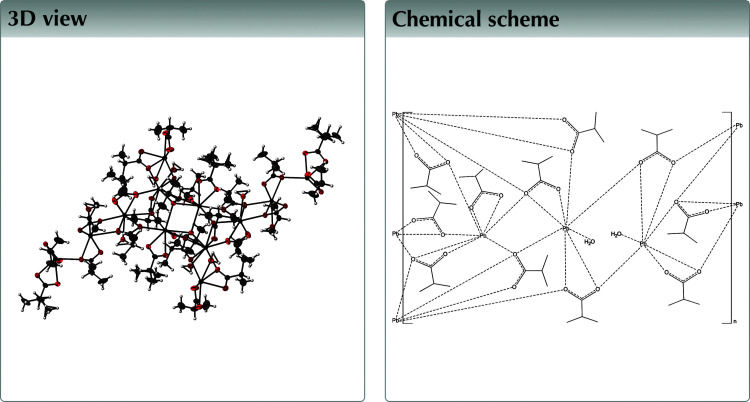

## Structure description

The structural features of metal carboxyl­ates, except formates and acetates, are strongly affected by voluminous hydro­phobic chains [*cf.* Duruz & Ubbelohde (1972 ▸) and Dumbleton & Lomer (1965 ▸)] which tend to be separated from the hydro­philic parts of these structures. The latter parts are composed of the cations, which are coordinated by the carboxyl­ate or water oxygen atoms. The hydro­philic parts can take the form of clustered aggregates, columns or planes, which then are surrounded by the hydro­phobic parts [see Samolová & Fábry (2020 ▸)]. In some cases there is a positional disorder of hydro­phobic chains realised, *e.g.* in barium dicalcium hexa­kis­(propano­ate) (Stadnicka & Glazer, 1980 ▸), or in the crystal structure of the title compound, [Pb_3_(C_4_H_7_O_2_)_6_(H_2_O)_2_]_*n*_.

The title structure has been determined previously by Fallon *et al.* (1997 ▸) without details regarding atomic coordin­ates and displacement parameters in the original publication. The current redetermination was undertaken because the deposited data in the Cambridge Structural Database (Groom *et al.*, 2016 ▸; version 5.41 from November 2019 with updates until August 2020), refcode REXBAX, is also incomplete. Here only atomic coordinates are given, and occupation factors of the disordered hydro­carbon chains are missing as well. In general, the quality of the study by Fallon *et al.* (1997 ▸) with a reliability factor *R* = 0.071, *wR* = 0.092 is below current standards. For example, the differences between the positions of the corresponding atoms in the original and the preset study is as large as 0.3 Å. However, it should be taken into account that the re-refined structure is based on data measured at 120 K with all non-H atoms refined with anisotropic displacement parameters compared to the previous determination at 193 K.

**Figure 1:**
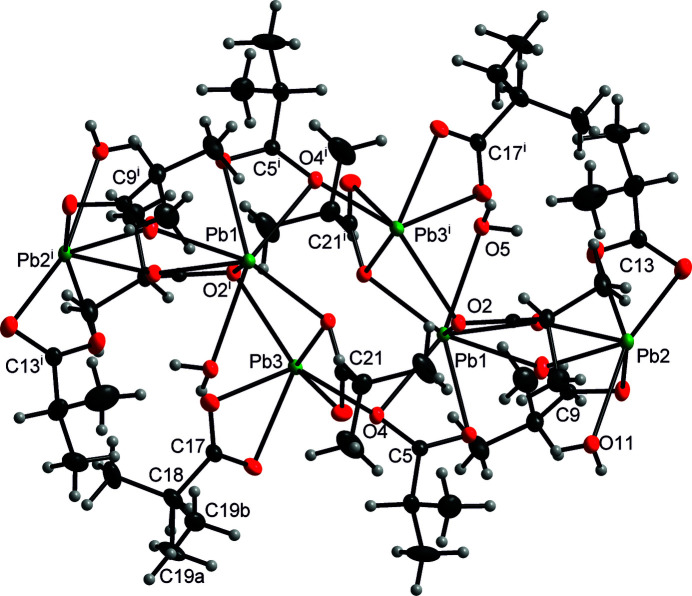
View of the core motif in the title structure showing the environments of the cations. Displacement ellipsoids of the Pb (dark green), O (red) and C (grey) atoms are shown at the 30% probability level while H atoms are shown as spheres of arbitrary radius. The positional disorder is shown. This involves the groups attached to C17 and C17^i^. Three terminal methyl groups are present.

There are three independent cations Pb1^2+^, Pb2^2+^ and Pb3^2+^ in the crystal structure. They are coordinated by the carboxyl­ate or water oxygen atoms, resulting in coordination numbers of [7 + 1], [6 + 1] and [5 + 3] for Pb1^2+^, Pb2^2+^ and Pb3^2+^, respectively. The coordination of each cation is irregular, suggesting stereoactivitity of the electron inert pair 6*s* ^2^. The coordination spheres of Pb1^2+^ and Pb2^2+^ include two and one coordinating carboxyl­ate groups in a bidentate bridging mode while Pb3^2+^ is coordinated in a simple bidentate mode. Each of the cations Pb1^2+^ and Pb2^2+^ is coordinated by one water mol­ecule. The corresponding Pb—O bond lengths are listed in Table 1 ▸. The bond-valence sums (Brese & O’Keeffe, 1991 ▸) of the cations are 1.977 (4), 2.115 (6) and 2.032 (5) valence units for Pb1^2+^, Pb2^2+^ and Pb3^2+^, respectively. The core of the structure is an eight-membered centrosymmetric ring composed of the atoms Pb1\O2^i^\Pb3\O4\Pb1\O2\Pb3^i^\O4^i^ (Fig. 1 ▸) [symmetry code (i): −*x* + 1, −*y* + 1, −*z* + 1]. Symmetry-equivalent Pb2^2+^ cations including their coordin­ating mol­ecules are attached to this core.

**Figure 2:**
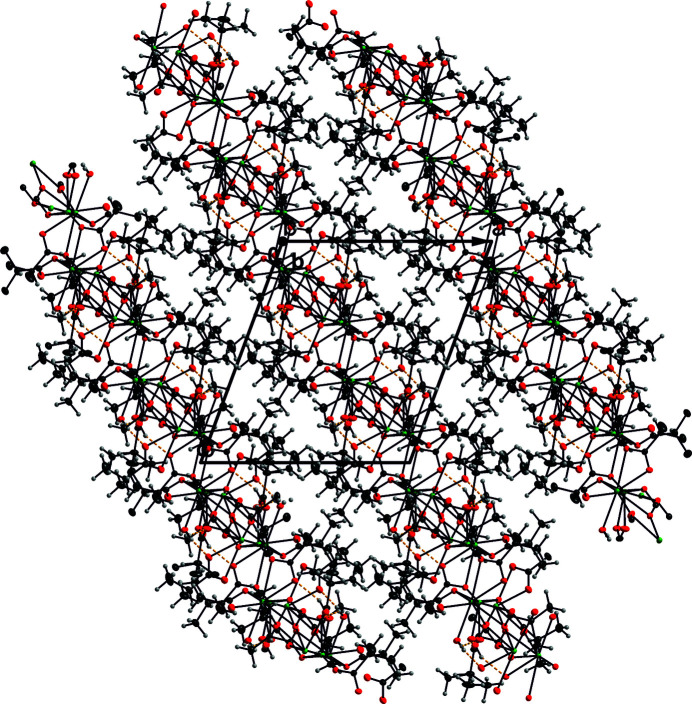
View of the ( 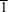 01) sheets in a view along the *b* axis. Displacement ellipsoids of the Pb, O and C atoms are shown at the 50% probability level while H atoms are shown as spheres of arbitrary radius. O—H⋯O hydrogen bonds are shown as dashed yellow lines; colour codes are as in Fig. 1 ▸.

The cations, carboxyl­ate oxygen atoms and water mol­ecules form the hydro­philic part of the structure that is characterized by sheets parallel to ( 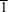 01) (Fig. 2 ▸). Each of the water mol­ecules is involved in an O_water_—H⋯O hydrogen bond of moderate strength (Gilli & Gilli, 2009 ▸) within a sheet (Table 2 ▸). These sheets are surrounded by hydro­phobic layers composed of 2-methyl­propanoic chains. Two methyl groups centered on the atoms C3 and C12 are protruding into the cation–oxygen sheet. The methyl group C19 is disordered over two sets of sites (split into C19*a* and C19*b*). The distances C_meth­yl_⋯C_meth­yl_ or C_methane­tri­yl_⋯C_meth­yl_ indicate the presence of van der Waals inter­actions. The shortest distance of this kind regards the contact C3⋯C10(−*x* + 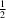 , *y* − 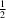 , −*z* + 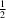 ) and equals 3.713 (5) Å.

## Synthesis and crystallization

The title structure was prepared by by disolution of 1.18 g of PbCO_3_ in a water solution (100 ml) of 0.78 g of 2-methyl­propanoic acid (molar ratio 1:2). The pH of the solution was adjusted to ∼6 by adding 2-methyl­propanoic acid. The solution was then filtered and concentrated at 313 K. After a crust had started to appear on the surface of the solution, heating was stopped and elongated colourless crystals appeared.

## Refinement

Crystal data, data collection and structure refinement details are summarized in Table 3 ▸. One of the 2-methyl­propano­ate anions involving atoms C18 and C19 and their attached hydrogen atoms is disordered in a 0.535 (9):0.465 (9) ratio. This disorder leads to a (virtual) distance C19*b*⋯C19*b*(–*x* + 1, –*y* + 1, –*z* + 2) of 2.358 (14) Å. A B-C type 1 Lorentzian isotropic (Becker & Coppens, 1974 ▸) extinction correction was applied.

## Supplementary Material

Crystal structure: contains datablock(s) global, I. DOI: 10.1107/S2414314620013115/wm4140sup1.cif

Structure factors: contains datablock(s) I. DOI: 10.1107/S2414314620013115/wm4140Isup2.hkl

Supporting information file. DOI: 10.1107/S2414314620013115/wm4140Isup3.smi

CCDC reference: 2034493

Additional supporting information:  crystallographic information; 3D view; checkCIF report

## Figures and Tables

**Table 1:** Selected bond lengths (Å)

Pb1—O1	2.5508 (19)	Pb2—O9	2.690 (3)
Pb1—O2	2.475 (2)	Pb2—O10	2.389 (3)
Pb1—O3	2.555 (3)	Pb2—O11	2.721 (3)
Pb1—O4	2.650 (2)	Pb3—O2^ii^	2.843 (2)
Pb1—O5	2.692 (3)	Pb3—O4	2.566 (2)
Pb1—O7	2.766 (2)	Pb3—O8^i^	2.834 (2)
Pb1—O9^i^	2.949 (2)	Pb3—O12	2.519 (2)
Pb1—O14^ii^	2.586 (2)	Pb3—O13	2.485 (2)
Pb2—O1	2.534 (2)	Pb3—O14	2.712 (2)
Pb2—O4^iii^	2.941 (2)	Pb3—O14^ii^	2.947 (2)
Pb2—O7	2.407 (2)	Pb3—O15	2.401 (3)
Pb2—O8	2.487 (2)		

Symmetry codes: (i) 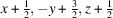 ; (ii) 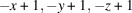 ; (iii) 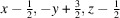 .

**Table 2:** Hydrogen-bond geometry (Å, °)

*D*—H⋯*A*	*D*—H	H⋯*A*	*D*⋯*A*	*D*—H⋯*A*
O5—H1*o*5⋯O10	0.84 (3)	1.95 (3)	2.788 (3)	171 (4)
O5—H2*o*5⋯O13^ii^	0.83 (3)	2.08 (4)	2.788 (4)	144 (4)
O11—H1*o*11⋯O3	0.84 (3)	2.06 (3)	2.859 (3)	157 (3)
O11—H2*o*11⋯O12^iii^	0.837 (16)	1.944 (17)	2.739 (3)	158 (4)

Symmetry codes: (ii) 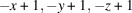 ; (iii) 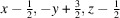 .

**Table 3:** Experimental details

Crystal data	
Chemical formula	[Pb_3_(C_4_H_7_O_2_)_6_(H_2_O)_2_]
*M*_r_	1180.2
Crystal system, space group	Monoclinic, *P*2_1_/*n*
Temperature (K)	120
*a*, *b*, *c* (Å)	12.7476 (4), 20.3424 (7), 14.3958 (4)
β (°)	110.329 (1)
*V* (Å^3^)	3500.55 (19)
*Z*	4
Radiation type	Mo *K*α
μ (mm^−1^)	14.45
Crystal size (mm)	0.25 × 0.19 × 0.16

Data collection	
Diffractometer	Bruker D8 VENTURE Kappa Duo PHOTON 100 CMOS
Absorption correction	Multi-scan (*SADABS*; Bruker, 2017 ▸)
*T*_min_, *T*_max_	0.123, 0.203
No. of measured, independent and observed [*I* > 3σ(*I*)] reflections	38736, 8019, 7372
*R*_int_	0.028
(sin θ/λ)_max_ (Å^−1^)	0.650

Refinement	
*R*[*F* > 3σ(*F*)], *wR*(*F*), *S*	0.016, 0.045, 1.25
No. of reflections	8019
No. of parameters	393
No. of restraints	4
H-atom treatment	H atoms treated by a mixture of independent and constrained refinement
Δρ_max_, Δρ_min_ (e Å^−3^)	0.89, −0.45

Computer programs: *APEX3* and *SAINT* (Bruker, 2017 ▸), *SHELXT* (Sheldrick, 2015 ▸), *JANA2006* (Petříček *et al.*, 2014 ▸) and *DIAMOND* (Brandenburg, 2015 ▸).

## full crystallographic data

### Crystal data

**Table:**

[Pb_3_(C_4_H_7_O_2_)_6_(H_2_O)_2_]	*F*(000) = 2192
*M**_r_* = 1180.2	*D*_x_ = 2.239 Mg m^−^^3^
Monoclinic, *P*2_1_/*n*	Mo *K*α radiation, λ = 0.71073 Å
Hall symbol: -P 2yn	Cell parameters from 9196 reflections
*a* = 12.7476 (4) Å	θ = 2.3–27.5°
*b* = 20.3424 (7) Å	µ = 14.45 mm^−^^1^
*c* = 14.3958 (4) Å	*T* = 120 K
β = 110.329 (1)°	Prism, colourless
*V* = 3500.55 (19) Å^3^	0.25 × 0.19 × 0.16 mm
*Z* = 4	

### Data collection

**Table:**

Bruker D8 VENTURE Kappa Duo PHOTON 100 CMOS diffractometer	8019 independent reflections
Radiation source: X-ray tube	7372 reflections with *I* > 3σ(*I*)
Quazar Mo multilayer optic monochromator	*R*_int_ = 0.028
φ and ω scans	θ_max_ = 27.5°, θ_min_ = 2.0°
Absorption correction: multi-scan (*SADABS*; Bruker, 2017)	*h* = −16→16
*T*_min_ = 0.123, *T*_max_ = 0.203	*k* = −25→26
38736 measured reflections	*l* = −18→18

### Refinement

**Table:**

Refinement on *F*^2^	Primary atom site location: dual
*R*[*F* > 3σ(*F*)] = 0.016	H atoms treated by a mixture of independent and constrained refinement
*wR*(*F*) = 0.045	Weighting scheme based on measured s.u.'s *w* = 1/(σ^2^(*I*) + 0.0004*I*^2^)
*S* = 1.25	(Δ/σ)_max_ = 0.031
8019 reflections	Δρ_max_ = 0.89 e Å^−^^3^
393 parameters	Δρ_min_ = −0.45 e Å^−^^3^
4 restraints	Extinction correction: B-C type 1 Lorentzian isotropic (Becker & Coppens, 1974)
197 constraints	Extinction coefficient: 1570 (140)

### Special details

**Table:**

Refinement. The non-hydrogen atoms were determined by SHELXT (Sheldrick, 2015). The methanetriyl hydrogen was placed into the calculated positions and refined under the following constraints: Cmethanetriyl—Hmethanetriyl = 1.00?Å, Uiso(Hmethanetriyl) = 1.2Ueq(Cmethanetriyl). After the anisotropic refinement of the non-hydrogen atoms with the methanetriyl hydrogen had been carried out the difference electron density map revealed other hydrogens. These hydrogens were refined under the following constraints: Cmethyl—Hmethyl = 0.98?Å, Uiso(Hmethyl) = 1.5Ueq(Cmethyl). The water hydrogen were refined using the distance restraints Owater—Hwater = 0.840?(1)?Å and the constraints Uiso(Hwater) = 1.5Ueq(Owater). The occupancies regarding the atoms C19a and C19b were treated in such a way that their sum equalled to 1 while the occupational parameter of C19a was refined. The attached hydrogens tothe atoms C19a and C19b were assigned the pertinent occupancies. The same holds for the methanetriyl hydrogens H1C18 and H1C18d which were assigned the occupancies of the atoms C19a and C19b, respectively. For the treatment of the disorder a dummy atom C18d was introduced with the same positional and displacement parameters as the atom C18.

### Fractional atomic coordinates and isotropic or equivalent isotropic displacement parameters (Å^2^)

**Table:**

	*x*	*y*	*z*	*U*_iso_*/*U*_eq_	Occ. (<1)
Pb1	0.457931 (9)	0.666292 (5)	0.371803 (7)	0.02067 (4)	
O1	0.25580 (17)	0.64686 (10)	0.25990 (15)	0.0251 (7)	
O2	0.33105 (18)	0.57622 (10)	0.38028 (15)	0.0260 (7)	
C1	0.2472 (3)	0.59615 (14)	0.3084 (2)	0.0233 (9)	
C2	0.1372 (3)	0.56115 (15)	0.2846 (2)	0.0277 (10)	
H1c2	0.151482	0.513523	0.301464	0.0332*	
C3	0.0685 (3)	0.56400 (18)	0.1738 (3)	0.0424 (13)	
H1c3	−0.005525	0.544768	0.162031	0.0636*	
H2c3	0.060016	0.609876	0.151671	0.0636*	
H3c3	0.106919	0.539167	0.136809	0.0636*	
C4	0.0735 (3)	0.5909 (2)	0.3467 (3)	0.0446 (14)	
H1c4	0.0019	0.568118	0.332205	0.0669*	
H2c4	0.117947	0.586115	0.41716	0.0669*	
H3c4	0.059878	0.637675	0.33056	0.0669*	
O3	0.33925 (19)	0.71358 (11)	0.46690 (15)	0.0309 (8)	
O4	0.48717 (18)	0.66048 (10)	0.56310 (15)	0.0258 (7)	
C5	0.3944 (3)	0.69003 (15)	0.5495 (2)	0.0253 (10)	
C6	0.3521 (3)	0.69758 (19)	0.6353 (2)	0.0355 (12)	
H1c6	0.402434	0.67256	0.693541	0.0425*	
C7	0.2359 (4)	0.6692 (2)	0.6102 (3)	0.0514 (17)	
H1c7	0.236512	0.622828	0.591746	0.0771*	
H2c7	0.212292	0.672676	0.667913	0.0771*	
H3c7	0.183636	0.693576	0.554601	0.0771*	
C8	0.3536 (5)	0.7691 (2)	0.6619 (4)	0.068 (2)	
H1c8	0.429831	0.786272	0.678983	0.1018*	
H2c8	0.303268	0.793569	0.605299	0.1018*	
H3c8	0.328773	0.774065	0.718739	0.1018*	
O5	0.4582 (2)	0.62074 (12)	0.19635 (17)	0.0333 (8)	
H1o5	0.409 (3)	0.6436 (18)	0.154 (2)	0.0499*	
H2o5	0.425 (3)	0.5866 (13)	0.171 (3)	0.0499*	
Pb2	0.166963 (9)	0.743253 (5)	0.148948 (8)	0.02364 (4)	
O7	0.3473 (2)	0.77396 (11)	0.26476 (17)	0.0345 (8)	
O8	0.25242 (19)	0.85471 (11)	0.17366 (16)	0.0326 (8)	
C9	0.3371 (3)	0.83431 (15)	0.2433 (2)	0.0263 (10)	
C10	0.4267 (3)	0.88229 (16)	0.3004 (2)	0.0335 (11)	
H1c10	0.390763	0.925582	0.303262	0.0402*	
C11	0.5087 (3)	0.8909 (2)	0.2468 (3)	0.0546 (17)	
H1c11	0.566209	0.922756	0.282494	0.082*	
H2c11	0.544104	0.84854	0.24371	0.082*	
H3c11	0.469125	0.906836	0.179461	0.082*	
C12	0.4841 (4)	0.8619 (2)	0.4079 (3)	0.0500 (15)	
H1c12	0.537211	0.896055	0.443059	0.075*	
H2c12	0.427875	0.855916	0.439399	0.075*	
H3c12	0.524265	0.820424	0.41043	0.075*	
O9	0.1781 (2)	0.77211 (13)	−0.02971 (18)	0.0376 (9)	
O10	0.2935 (2)	0.70285 (14)	0.07275 (18)	0.0423 (10)	
C13	0.2617 (3)	0.73523 (17)	−0.0076 (2)	0.0312 (11)	
C14	0.3287 (3)	0.7291 (2)	−0.0759 (3)	0.0444 (15)	
H1c14	0.288158	0.752046	−0.139698	0.0533*	
C15	0.3419 (4)	0.6578 (2)	−0.1001 (3)	0.0592 (19)	
H1c15	0.380099	0.655464	−0.148479	0.0888*	
H2c15	0.386195	0.634583	−0.039544	0.0888*	
H3c15	0.267955	0.637364	−0.127909	0.0888*	
C16	0.4430 (5)	0.7606 (3)	−0.0257 (5)	0.075 (3)	
H1c16	0.483707	0.761903	−0.072224	0.1132*	
H2c16	0.433209	0.805392	−0.005155	0.1132*	
H3c16	0.485564	0.734628	0.032608	0.1132*	
O11	0.1370 (2)	0.76307 (12)	0.32500 (18)	0.0323 (8)	
H1o11	0.188 (3)	0.7521 (19)	0.3781 (19)	0.0485*	
H2o11	0.135 (4)	0.8038 (7)	0.333 (3)	0.0485*	
Pb3	0.579586 (10)	0.550258 (5)	0.631759 (8)	0.02294 (4)	
O12	0.6090 (2)	0.60407 (11)	0.79647 (16)	0.0343 (8)	
O13	0.6471 (2)	0.49866 (12)	0.79861 (17)	0.0397 (9)	
C17	0.6397 (3)	0.55072 (16)	0.8429 (2)	0.0320 (11)	
C18	0.6643 (4)	0.5484 (2)	0.9539 (3)	0.0458 (15)	
H1c18	0.594513	0.528771	0.958846	0.055*	0.535 (9)
H1c18d	0.6843	0.593414	0.982517	0.055*	0.465 (9)
C19a	0.6750 (9)	0.6088 (4)	1.0031 (5)	0.061 (4)	0.535 (9)
H1c19a	0.677693	0.601374	1.071125	0.0912*	0.535 (9)
H2c19a	0.743997	0.630557	1.004193	0.0912*	0.535 (9)
H3c19a	0.610676	0.636753	0.968277	0.0912*	0.535 (9)
C19b	0.5653 (7)	0.5273 (5)	0.9724 (6)	0.046 (3)	0.465 (9)
H1c19b	0.578329	0.530232	1.043463	0.0692*	0.465 (9)
H2c19b	0.502285	0.555631	0.93591	0.0692*	0.465 (9)
H3c19b	0.54815	0.481722	0.950328	0.0692*	0.465 (9)
C20	0.7655 (4)	0.5051 (3)	1.0056 (3)	0.071 (2)	
H1c20	0.83205	0.524098	0.996681	0.1058*	
H2c20	0.776976	0.502605	1.076405	0.1058*	
H3c20	0.752465	0.460921	0.976808	0.1058*	
O14	0.44049 (18)	0.44519 (10)	0.57833 (16)	0.0260 (7)	
O15	0.4044 (2)	0.53142 (12)	0.65501 (18)	0.0369 (9)	
C21	0.3826 (3)	0.47358 (16)	0.6224 (2)	0.0277 (10)	
C22	0.2840 (3)	0.43782 (18)	0.6344 (3)	0.0374 (13)	
H1c22	0.302803	0.390086	0.645942	0.0449*	
C23	0.1847 (4)	0.4454 (3)	0.5401 (3)	0.067 (2)	
H1c23	0.118855	0.425061	0.548466	0.0998*	
H2c23	0.170189	0.492206	0.525034	0.0998*	
H3c23	0.200537	0.423821	0.485533	0.0998*	
C24	0.2598 (4)	0.4594 (3)	0.7239 (3)	0.074 (2)	
H1c24	0.208577	0.427987	0.737464	0.1117*	
H2c24	0.329677	0.461249	0.780843	0.1117*	
H3c24	0.22513	0.503021	0.712105	0.1117*	

### Atomic displacement parameters (Å^2^)

**Table:**

	*U*^11^	*U*^22^	*U*^33^	*U*^12^	*U*^13^	*U*^23^
Pb1	0.01967 (6)	0.02025 (6)	0.02043 (6)	0.00001 (4)	0.00487 (4)	0.00093 (4)
O1	0.0232 (11)	0.0218 (10)	0.0290 (10)	−0.0017 (8)	0.0074 (9)	0.0032 (8)
O2	0.0233 (11)	0.0253 (11)	0.0264 (10)	−0.0001 (9)	0.0050 (9)	0.0029 (9)
C1	0.0245 (15)	0.0218 (14)	0.0234 (13)	0.0009 (11)	0.0080 (12)	−0.0036 (11)
C2	0.0233 (15)	0.0224 (15)	0.0326 (16)	−0.0044 (12)	0.0036 (13)	0.0026 (12)
C3	0.036 (2)	0.037 (2)	0.040 (2)	−0.0076 (16)	−0.0036 (16)	0.0017 (15)
C4	0.0301 (19)	0.049 (2)	0.058 (2)	−0.0080 (16)	0.0205 (18)	−0.0003 (19)
O3	0.0316 (12)	0.0364 (12)	0.0236 (10)	0.0102 (10)	0.0084 (10)	0.0025 (9)
O4	0.0221 (11)	0.0274 (11)	0.0264 (10)	0.0021 (8)	0.0064 (9)	0.0032 (8)
C5	0.0252 (15)	0.0236 (14)	0.0258 (14)	−0.0020 (12)	0.0073 (12)	−0.0022 (12)
C6	0.0254 (16)	0.055 (2)	0.0256 (15)	0.0030 (15)	0.0086 (13)	0.0017 (15)
C7	0.040 (2)	0.067 (3)	0.055 (2)	−0.0048 (19)	0.026 (2)	0.001 (2)
C8	0.078 (4)	0.074 (3)	0.071 (3)	−0.021 (3)	0.051 (3)	−0.040 (3)
O5	0.0315 (13)	0.0375 (13)	0.0267 (11)	0.0068 (10)	0.0049 (10)	0.0009 (10)
Pb2	0.02020 (6)	0.02509 (6)	0.02363 (6)	0.00152 (4)	0.00507 (5)	0.00357 (4)
O7	0.0282 (12)	0.0241 (11)	0.0420 (13)	−0.0025 (9)	0.0005 (11)	0.0073 (10)
O8	0.0269 (12)	0.0310 (12)	0.0349 (12)	0.0035 (10)	0.0043 (10)	0.0062 (10)
C9	0.0256 (16)	0.0271 (16)	0.0275 (15)	0.0011 (12)	0.0109 (13)	0.0011 (12)
C10	0.0334 (18)	0.0244 (16)	0.0372 (17)	−0.0017 (13)	0.0053 (15)	0.0021 (13)
C11	0.040 (2)	0.068 (3)	0.057 (2)	−0.022 (2)	0.018 (2)	−0.007 (2)
C12	0.054 (3)	0.045 (2)	0.0380 (19)	−0.0101 (19)	−0.0005 (18)	−0.0022 (17)
O9	0.0356 (13)	0.0421 (14)	0.0374 (13)	0.0107 (11)	0.0157 (11)	0.0136 (11)
O10	0.0374 (14)	0.0579 (17)	0.0367 (13)	0.0189 (12)	0.0195 (12)	0.0186 (12)
C13	0.0309 (17)	0.0362 (18)	0.0271 (15)	0.0020 (14)	0.0109 (14)	0.0057 (13)
C14	0.044 (2)	0.058 (2)	0.041 (2)	0.0143 (18)	0.0269 (18)	0.0174 (18)
C15	0.065 (3)	0.074 (3)	0.044 (2)	0.000 (2)	0.026 (2)	−0.014 (2)
C16	0.070 (3)	0.066 (3)	0.115 (5)	−0.019 (3)	0.064 (4)	−0.011 (3)
O11	0.0322 (13)	0.0293 (12)	0.0319 (12)	0.0059 (10)	0.0067 (10)	0.0014 (10)
Pb3	0.02559 (6)	0.02066 (6)	0.01982 (6)	0.00002 (4)	0.00442 (5)	0.00007 (4)
O12	0.0439 (14)	0.0293 (12)	0.0254 (11)	−0.0016 (10)	0.0067 (10)	−0.0043 (9)
O13	0.0575 (17)	0.0326 (13)	0.0265 (11)	0.0107 (12)	0.0115 (12)	0.0047 (10)
C17	0.0340 (18)	0.0351 (18)	0.0239 (15)	0.0019 (14)	0.0062 (14)	0.0005 (13)
C18	0.047 (2)	0.064 (3)	0.0238 (17)	0.0024 (19)	0.0093 (16)	−0.0017 (16)
C19a	0.108 (8)	0.042 (4)	0.028 (3)	0.001 (5)	0.018 (4)	−0.010 (3)
C19b	0.036 (4)	0.071 (6)	0.036 (4)	0.004 (4)	0.018 (4)	−0.005 (4)
C20	0.052 (3)	0.127 (5)	0.0273 (19)	0.021 (3)	0.0073 (19)	0.015 (2)
O14	0.0241 (11)	0.0273 (11)	0.0258 (11)	0.0025 (8)	0.0077 (9)	0.0024 (8)
O15	0.0378 (14)	0.0328 (13)	0.0425 (13)	−0.0034 (11)	0.0168 (12)	−0.0082 (11)
C21	0.0269 (16)	0.0302 (16)	0.0250 (14)	0.0009 (13)	0.0077 (13)	0.0033 (12)
C22	0.0310 (18)	0.040 (2)	0.046 (2)	−0.0015 (15)	0.0188 (17)	0.0040 (16)
C23	0.040 (2)	0.112 (5)	0.042 (2)	−0.031 (3)	0.007 (2)	−0.004 (2)
C24	0.043 (3)	0.146 (5)	0.042 (2)	−0.025 (3)	0.025 (2)	−0.011 (3)

### Geometric parameters (Å, º)

**Table:**

Pb1—O1	2.5508 (19)	C11—H2c11	0.98
Pb1—O2	2.475 (2)	C11—H3c11	0.98
Pb1—O3	2.555 (3)	C12—H1c12	0.98
Pb1—O4	2.650 (2)	C12—H2c12	0.98
Pb1—O5	2.692 (3)	C12—H3c12	0.98
Pb1—O7	2.766 (2)	O9—C13	1.250 (4)
Pb1—O9^i^	2.949 (2)	O10—C13	1.268 (4)
Pb1—O14^ii^	2.586 (2)	C13—C14	1.516 (6)
Pb2—O1	2.534 (2)	C14—H1c14	1
Pb2—O4^iii^	2.941 (2)	C14—C15	1.513 (6)
Pb2—O7	2.407 (2)	C14—C16	1.524 (7)
Pb2—O8	2.487 (2)	C15—H1c15	0.98
Pb2—O9	2.690 (3)	C15—H2c15	0.98
Pb2—O10	2.389 (3)	C15—H3c15	0.98
Pb2—O11	2.721 (3)	C16—H1c16	0.98
Pb3—O2^ii^	2.843 (2)	C16—H2c16	0.98
Pb3—O4	2.566 (2)	C16—H3c16	0.98
Pb3—O8^i^	2.834 (2)	O11—H1o11	0.84 (3)
Pb3—O12	2.519 (2)	O11—H2o11	0.837 (16)
Pb3—O13	2.485 (2)	H1o11—H2o11	1.29 (4)
Pb3—O14	2.712 (2)	O12—C17	1.263 (4)
Pb3—O14^ii^	2.947 (2)	O13—C17	1.257 (4)
Pb3—O15	2.401 (3)	C17—C18	1.518 (5)
O1—C1	1.271 (4)	C18—H1c18	1
O2—C1	1.269 (3)	C18—H1c18d	1
C1—C2	1.503 (4)	C18—C19a	1.401 (9)
C2—H1c2	1	C18—C19b	1.442 (11)
C2—C3	1.531 (5)	C18—C20	1.525 (6)
C2—C4	1.526 (6)	H1c18—H1c18d	1.6983
C3—H1c3	0.98	H1c18—H1c19b	1.3046
C3—H2c3	0.98	H1c18—H2c19b	1.2329
C3—H3c3	0.98	H1c18—H3c19b	1.1091
C4—H1c4	0.98	H1c18d—H1c19a	1.3172
C4—H2c4	0.98	H1c18d—H2c19a	1.0404
C4—H3c4	0.98	H1c18d—H3c19a	1.2516
O3—C5	1.247 (3)	C19a—H1c19a	0.98
O4—C5	1.280 (4)	C19a—H2c19a	0.98
C5—C6	1.518 (5)	C19a—H3c19a	0.98
C6—H1c6	1	C19b—H1c19b	0.98
C6—C7	1.512 (6)	C19b—H2c19b	0.98
C6—C8	1.503 (6)	C19b—H3c19b	0.98
C7—H1c7	0.98	C20—H1c20	0.98
C7—H2c7	0.98	C20—H2c20	0.98
C7—H3c7	0.98	C20—H3c20	0.98
C8—H1c8	0.98	O14—C21	1.267 (5)
C8—H2c8	0.98	O15—C21	1.262 (4)
C8—H3c8	0.98	C21—C22	1.513 (5)
O5—H1o5	0.84 (3)	C22—H1c22	1
O5—H2o5	0.83 (3)	C22—C23	1.508 (5)
H1o5—H2o5	1.19 (5)	C22—C24	1.491 (7)
O7—C9	1.262 (4)	C23—H1c23	0.98
O8—C9	1.261 (3)	C23—H2c23	0.98
C9—C10	1.509 (4)	C23—H3c23	0.98
C10—H1c10	1	C24—H1c24	0.98
C10—C11	1.510 (7)	C24—H2c24	0.98
C10—C12	1.522 (5)	C24—H3c24	0.98
C11—H1c11	0.98		
			
O1—Pb1—O2	51.86 (6)	C5—C6—C7	111.2 (3)
O1—Pb1—O3	74.96 (7)	C5—C6—C8	109.2 (4)
O1—Pb1—O4	113.32 (7)	H1c6—C6—C7	107.22
O1—Pb1—O5	71.61 (8)	H1c6—C6—C8	109.35
O1—Pb1—O7	64.11 (7)	C7—C6—C8	110.9 (4)
O1—Pb1—O9^i^	162.47 (7)	C6—C7—H1c7	109.47
O1—Pb1—O14^ii^	109.75 (6)	C6—C7—H2c7	109.47
O2—Pb1—O3	74.55 (8)	C6—C7—H3c7	109.47
O2—Pb1—O4	76.95 (7)	H1c7—C7—H2c7	109.47
O2—Pb1—O5	90.31 (8)	H1c7—C7—H3c7	109.47
O2—Pb1—O7	113.48 (7)	H2c7—C7—H3c7	109.47
O2—Pb1—O9^i^	145.10 (6)	C6—C8—H1c8	109.47
O2—Pb1—O14^ii^	67.04 (7)	C6—C8—H2c8	109.47
O3—Pb1—O4	49.89 (6)	C6—C8—H3c8	109.47
O3—Pb1—O5	145.75 (7)	H1c8—C8—H2c8	109.47
O3—Pb1—O7	73.63 (8)	H1c8—C8—H3c8	109.47
O3—Pb1—O9^i^	102.97 (7)	H2c8—C8—H3c8	109.47
O3—Pb1—O14^ii^	120.93 (8)	O7—C9—O8	120.1 (3)
O4—Pb1—O5	156.09 (7)	O7—C9—C10	120.1 (2)
O4—Pb1—O7	118.34 (7)	O8—C9—C10	119.8 (3)
O4—Pb1—O9^i^	75.67 (7)	C9—C10—H1c10	108.76
O4—Pb1—O14^ii^	78.25 (7)	C9—C10—C11	108.9 (3)
O5—Pb1—O7	85.21 (7)	C9—C10—C12	112.5 (3)
O5—Pb1—O9^i^	106.62 (8)	H1c10—C10—C11	109.15
O5—Pb1—O14^ii^	78.16 (7)	H1c10—C10—C12	105.28
O7—Pb1—O9^i^	98.46 (7)	C11—C10—C12	112.1 (3)
O7—Pb1—O14^ii^	163.37 (8)	C10—C11—H1c11	109.47
O9^i^—Pb1—O14^ii^	86.44 (7)	C10—C11—H2c11	109.47
Pb1—O2—Pb3^ii^	112.60 (9)	C10—C11—H3c11	109.47
Pb1—O4—Pb2^i^	102.40 (8)	H1c11—C11—H2c11	109.47
Pb1—O4—Pb3	108.62 (8)	H1c11—C11—H3c11	109.47
O1—Pb2—O4^iii^	153.20 (7)	H2c11—C11—H3c11	109.47
O1—Pb2—O7	69.76 (7)	C10—C12—H1c12	109.47
O1—Pb2—O8	122.53 (6)	C10—C12—H2c12	109.47
O1—Pb2—O9	127.67 (8)	C10—C12—H3c12	109.47
O1—Pb2—O10	78.35 (8)	H1c12—C12—H2c12	109.47
O1—Pb2—O11	72.79 (7)	H1c12—C12—H3c12	109.47
O4^iii^—Pb2—O7	122.40 (7)	H2c12—C12—H3c12	109.47
O4^iii^—Pb2—O8	72.04 (6)	O9—C13—O10	121.2 (4)
O4^iii^—Pb2—O9	75.24 (7)	O9—C13—C14	120.4 (3)
O4^iii^—Pb2—O10	126.01 (8)	O10—C13—C14	118.4 (3)
O4^iii^—Pb2—O11	87.23 (7)	C13—C14—H1c14	109.21
O7—Pb2—O8	53.04 (7)	C13—C14—C15	111.2 (4)
O7—Pb2—O9	104.51 (8)	C13—C14—C16	108.6 (4)
O7—Pb2—O10	76.85 (9)	H1c14—C14—C15	107.43
O7—Pb2—O11	74.08 (9)	H1c14—C14—C16	110.07
O8—Pb2—O9	76.80 (8)	C15—C14—C16	110.3 (4)
O8—Pb2—O10	92.71 (9)	C14—C15—H1c15	109.47
O8—Pb2—O11	85.87 (8)	C14—C15—H2c15	109.47
O9—Pb2—O10	50.78 (8)	C14—C15—H3c15	109.47
O9—Pb2—O11	158.36 (7)	H1c15—C15—H2c15	109.47
O10—Pb2—O11	144.50 (7)	H1c15—C15—H3c15	109.47
Pb2—O8—Pb3^iii^	108.78 (8)	H2c15—C15—H3c15	109.47
Pb1^iii^—O9—Pb2	101.23 (9)	C14—C16—H1c16	109.47
O2^ii^—Pb3—O4	155.14 (7)	C14—C16—H2c16	109.47
O2^ii^—Pb3—O8^i^	109.38 (7)	C14—C16—H3c16	109.47
O2^ii^—Pb3—O12	121.26 (6)	H1c16—C16—H2c16	109.47
O2^ii^—Pb3—O13	70.44 (8)	H1c16—C16—H3c16	109.47
O2^ii^—Pb3—O14	60.39 (6)	H2c16—C16—H3c16	109.47
O2^ii^—Pb3—O14^ii^	81.93 (6)	H1o11—O11—H2o11	101 (4)
O2^ii^—Pb3—O15	106.01 (8)	O12—C17—O13	121.3 (3)
O4—Pb3—O8^i^	72.83 (6)	O12—C17—C18	119.8 (3)
O4—Pb3—O12	83.17 (7)	O13—C17—C18	118.9 (3)
O4—Pb3—O13	134.42 (8)	C17—C18—H1c18	102.94
O4—Pb3—O14	114.33 (6)	C17—C18—H1c18d	109.58
O4—Pb3—O14^ii^	73.30 (7)	C17—C18—C19a	116.9 (4)
O4—Pb3—O15	82.44 (8)	C17—C18—C19b	109.2 (4)
O8^i^—Pb3—O12	69.68 (8)	C17—C18—C20	111.2 (4)
O8^i^—Pb3—O13	95.73 (7)	C18—C19a—H1c19a	109.47
O8^i^—Pb3—O14	168.87 (8)	C18—C19a—H2c19a	109.47
O8^i^—Pb3—O14^ii^	88.91 (6)	C18—C19a—H3c19a	109.47
O8^i^—Pb3—O15	140.45 (8)	H1c18d—C19a—H1c19a	126.38
O12—Pb3—O13	52.07 (8)	H1c18d—C19a—H2c19a	83.68
O12—Pb3—O14	118.57 (8)	H1c18d—C19a—H3c19a	114.3
O12—Pb3—O14^ii^	152.14 (7)	H1c19a—C19a—H2c19a	109.47
O12—Pb3—O15	77.26 (9)	H1c19a—C19a—H3c19a	109.47
O13—Pb3—O14	85.07 (7)	H2c19a—C19a—H3c19a	109.47
O13—Pb3—O14^ii^	151.98 (8)	C18—C19b—H1c19b	109.47
O13—Pb3—O15	79.89 (9)	C18—C19b—H2c19b	109.47
O14—Pb3—O14^ii^	85.33 (7)	C18—C19b—H3c19b	109.47
O14—Pb3—O15	50.63 (8)	H1c19b—C19b—H2c19b	109.47
O14^ii^—Pb3—O15	113.25 (7)	H1c19b—C19b—H3c19b	109.47
Pb1^ii^—O14—Pb3	113.41 (7)	H2c19b—C19b—H3c19b	109.47
Pb1^ii^—O14—Pb3^ii^	99.73 (8)	C18—C20—H1c20	109.47
Pb3—O14—Pb3^ii^	94.67 (7)	C18—C20—H2c20	109.47
O1—C1—O2	119.9 (3)	C18—C20—H3c20	109.47
O1—C1—C2	120.7 (2)	H1c20—C20—H2c20	109.47
O2—C1—C2	119.3 (3)	H1c20—C20—H3c20	109.47
C1—C2—H1c2	109.09	H2c20—C20—H3c20	109.47
C1—C2—C3	111.8 (3)	O14—C21—O15	121.3 (3)
C1—C2—C4	108.8 (3)	O14—C21—C22	119.3 (3)
H1c2—C2—C3	106.26	O15—C21—C22	119.4 (3)
H1c2—C2—C4	109.33	C21—C22—H1c22	109.01
C3—C2—C4	111.5 (3)	C21—C22—C23	108.6 (3)
C2—C3—H1c3	109.47	C21—C22—C24	112.8 (3)
C2—C3—H2c3	109.47	H1c22—C22—C23	109.05
C2—C3—H3c3	109.47	H1c22—C22—C24	104.55
H1c3—C3—H2c3	109.47	C23—C22—C24	112.7 (4)
H1c3—C3—H3c3	109.47	C22—C23—H1c23	109.47
H2c3—C3—H3c3	109.47	C22—C23—H2c23	109.47
C2—C4—H1c4	109.47	C22—C23—H3c23	109.47
C2—C4—H2c4	109.47	H1c23—C23—H2c23	109.47
C2—C4—H3c4	109.47	H1c23—C23—H3c23	109.47
H1c4—C4—H2c4	109.47	H2c23—C23—H3c23	109.47
H1c4—C4—H3c4	109.47	C22—C24—H1c24	109.47
H2c4—C4—H3c4	109.47	C22—C24—H2c24	109.47
O3—C5—O4	120.7 (3)	C22—C24—H3c24	109.47
O3—C5—C6	119.7 (3)	H1c24—C24—H2c24	109.47
O4—C5—C6	119.6 (3)	H1c24—C24—H3c24	109.47
C5—C6—H1c6	108.97	H2c24—C24—H3c24	109.47

Symmetry codes: (i) *x*+1/2, −*y*+3/2, *z*+1/2; (ii) −*x*+1, −*y*+1, −*z*+1; (iii) *x*−1/2, −*y*+3/2, *z*−1/2.

### Hydrogen-bond geometry (Å, º)

**Table:**

*D*—H···*A*	*D*—H	H···*A*	*D*···*A*	*D*—H···*A*
O5—H1*o*5···O10	0.84 (3)	1.95 (3)	2.788 (3)	171 (4)
O5—H2*o*5···O13^ii^	0.83 (3)	2.08 (4)	2.788 (4)	144 (4)
O11—H1*o*11···O3	0.84 (3)	2.06 (3)	2.859 (3)	157 (3)
O11—H2*o*11···O12^iii^	0.837 (16)	1.944 (17)	2.739 (3)	158 (4)

Symmetry codes: (ii) −*x*+1, −*y*+1, −*z*+1; (iii) *x*−1/2, −*y*+3/2, *z*−1/2.
